# Editorial: Multi-target directed ligands for the treatment of cancer

**DOI:** 10.3389/fonc.2022.980141

**Published:** 2022-08-05

**Authors:** Margherita Brindisi, Sonja M. Kessler, Vinod Kumar, Clemens Zwergel

**Affiliations:** ^1^ Department of Pharmacy, University of Napoli Federico II, DoE Department of Excellence 2018–2022, Napoli, Italy; ^2^ Experimental Pharmacology for Natural Sciences, Institute of Pharmacy, Martin Luther University Halle-Wittenberg, Halle, Germany; ^3^ Department of Chemistry, Central University of Punjab, Bathinda, Punjab, India; ^4^ Department of Drug Chemistry & Technologies, Sapienza University of Rome, Rome, Italy

**Keywords:** anticancer agents, multi-target directed ligands (MTDLs), multifactorial disease, drug resistance, polypharmacology drugs

Cancer remains a major cause of mortality. However, significant progress has been made in understanding the molecular basis of the disease. Cancer is a multigenic and multicellular disease characterized by a multifactorial etiology, which initiates uncontrolled cell growth. The major problem associated with current cancer treatment regimens is ascribable to the development of resistance to therapy, which may arise due to several reasons, such as the overexpression of anti-apoptotic proteins, mutations in key signaling molecules, overexpression of drug efflux pumps, or the presence of dormant and/or resistant tumor cells. Most of the anticancer drugs in clinical use are based on the principle of ‘one molecule - one target - one malady’. However, multifactorial diseases such as cancer may greatly benefit from therapies simultaneously hitting multiple key pathways and/or their pathogenic cross-talk.

The design strategy for multi-target-directed ligands (MTDLs) involves the incorporation of two or more distinct pharmacophores of different drugs in a single structure to develop hybrid molecules. MTDLs can bind/inhibit two or more targets simultaneously, thus boosting the compound’s therapeutic potential *via* a polypharmacological approach. In addition, these also eliminate the chances of development of drug resistance frequently observed in case of single targeted regimens. Modern drug discovery has the power to identify potential multifunctional modulators for biologically and clinically validated targets among millions of compounds. The design of MTDLs is an essential and promising research area since recent research confirmed the potential therapeutic benefit for managing/treating complex multifactorial diseases. Despite the significant amount of drug discoveries in the vast field of cancer therapy, there is still an urgent need for novel and innovative treatments. The MTDL approach holds great potential in cancer therapy since it may significantly simplify treatment regimens with respect to standard combination therapy, reduce the risk of possible drug-drug interactions, and most importantly, limit the insurgence of resistance.

In the present Research Topic, several multitargeted approaches to cancer therapy are showcased in original articles and discussed from an interdisciplinary point of view, thus providing an updated picture of the latest progress in the field.

Lawal et al. applied computational and multi-omics studies to identify the targets of NSC765598, a novel small molecule derivative of salicylanilide. First, they screened the compound *in vitro* against the National Cancer Institute 60 (NCI60) human tumor cell lines and subsequently used specific molecular docking studies to identify potential ligand-protein interactions comparing the NSC765598 anticancer fingerprints with NCI standard agents. As a result, they identified the mammalian target of rapamycin (mTOR), the epidermal growth factor receptor (EGFR), the inducible nitric oxide synthase (iNOS), the mitogen-activated protein 2 kinase 1 (MAP2K1), the fibroblast growth factor receptor (FGFR), and the transforming growth factor-beta1 (TGFB1) as potential targets for NSC765598. In summary, NSC765598 displayed numerous potential multitarget properties and promising anticancer activities in several cancer cell lines, thus warranting further preclinical studies.

In their original research article, Yan et al. validated the *in vitro* efficacy on hematologic cells as well as the mechanism of action of a PI3K/HDAC dual-target inhibitor. The antiproliferative effects were also confirmed *in vivo* using EL4 and A20 xenograft models.

Wu et al. examined the effects of Xihuang pills (XHP), known in traditional Chinese medicine containing various herbs, on prostate cancer. The active principles of the various herbs in XHP were searched in specific publicly available databases, isolated, and subsequently tested on PC3 and LNCaP cells. Gene ontology and KEGG pathway analysis led to the identification of the PI3K/Akt/mTOR signaling pathway as primary targets of the XHP active principles. The dose-dependent effect on the expression levels of target proteins fosters deeper evaluation of this herbal mix.

Xiong et al. examined, in a clinical setting, the novel anti-angiogenesis compound anlotinib, a tyrosine kinase inhibitor that targets vascular endothelial growth factor receptors (VEGFRs), fibroblast growth factor receptors (FGFRs), and platelet-derived growth factor receptors (PDGFRs). The research team studied, in non-small cell lung cancer (NSCLC) patients, the influence of the body mass index (BMI) on the survival rate under anlotinib treatment. They revealed a U-shaped relationship between BMI and risk of death in patients receiving anlotinib for advanced NSCLC; thus, a high BMI, as well as a low one, can be associated with a worse survival rate.

Chen et al. analyzed Ganoderic acid Me (GA-Me), a natural bioactive compound derived from Ganoderma lucidum, *via* a whole-transcriptome sequencing approach to assess the long noncoding RNA (lncRNA), circular RNA (circRNA), microRNA (miRNA), and messenger RNA (mRNA) profiles in colorectal cancer (CRC). *Via* this approach, they identified matrix metalloprotease (MMP) 2 and MMP9 as potential targets and confirmed the binding of GA-Me to these enzymes *via* a docking approach. Furthermore, they were able to show that GA-Me inhibited proliferation, induced DNA fragmentation, and significantly activated caspase-9 and caspase-3 in HCT116 cells. In summary, their results suggested that GA-Me is a promising multitarget lead compound for CRC treatment.

Within this Research Topic, we have seen promising interdisciplinary workflows supporting the implementation of multitarget approaches in cancer biology and treatment. All contributions to this Research Topic strengthen the link between the development of multi-target directed ligands and oncology, showing a high grade of innovation and, at the same time, significant translational potential towards preclinical or even clinical applications in numerous types of cancer.

## Author contributions

All the authors have contributed to the composition and the revision of this Editorial Article.

## Conflict of interest

The authors declare that the research was conducted in the absence of any commercial or financial relationships that could be construed as a potential conflict of interest.

## Publisher’s note

All claims expressed in this article are solely those of the authors and do not necessarily represent those of their affiliated organizations, or those of the publisher, the editors and the reviewers. Any product that may be evaluated in this article, or claim that may be made by its manufacturer, is not guaranteed or endorsed by the publisher.

